# A Comparative Assessment of ChatGPT, Gemini, and DeepSeek Accuracy: Examining Visual Medical Assessment in Internal Medicine Cases with and Without Clinical Context

**DOI:** 10.3390/diagnostics16030388

**Published:** 2026-01-26

**Authors:** Rayah Asiri, Azfar Athar Ishaqui, Salman Ashfaq Ahmad, Muhammad Imran, Khalid Orayj, Adnan Iqbal

**Affiliations:** 1 Department of Clinical Pharmacy, College of Pharmacy, King Khalid University, Abha 62583, Saudi Arabia; 2 Faculty of Pharmacy, Iqra University, Karachi 75850, Pakistan; 3 Department of Pharmacology, Faculty of Pharmacy and Pharmaceutical Sciences, University of Karachi, Karachi 75270, Pakistan

**Keywords:** large language models, medical image interpretation, clinical history, internal medicine imaging, differential diagnosis agreement

## Abstract

**Background and Aim:** Large language models (LLMs) demonstrate significant potential in assisting with medical image interpretation. However, the diagnostic accuracy of general-purpose LLMs on image-based internal medicine cases and the added value of brief clinical history remain unclear. This study evaluated three general-purpose LLMs (ChatGPT, Gemini, and DeepSeek) on expert-curated cases to quantify diagnostic accuracy with image-only input versus image plus brief clinical context. **Methods:** We conducted a comparative evaluation using 138 expert-curated cases from Harrison’s Visual Case Challenge. Each case was presented to the models in two distinct phases: Phase 1 (image only) and Phase 2 (image plus a brief clinical history). The primary endpoint was top-1 diagnostic accuracy for the textbook diagnosis, comparing performance with versus without a brief clinical history. Secondary/Exploratory analyses compared models and assessed agreement between model-generated differential lists and the textbook differential. Statistical analysis included Wilson 95% confidence intervals, McNemar’s tests, Cochran’s Q with Benjamini–Hochberg correction, and Wilcoxon signed-rank tests. **Results:** The inclusion of clinical history substantially improved diagnostic accuracy for all models. ChatGPT’s accuracy increased from 50.7% in Phase 1 to 80.4% in Phase 2. Gemini’s accuracy improved from 39.9% to 72.5%, and DeepSeek’s accuracy rose from 30.4% to 75.4%. In Phase 2, diagnostic accuracy reached at least 65% across most disease nature and organ system categories. However, agreement with the reference differential diagnoses remained modest, with average overlap rates of 6.99% for ChatGPT, 36.39% for Gemini, and 32.74% for DeepSeek. **Conclusions:** The provision of brief clinical history significantly enhances the diagnostic accuracy of large language models on visual internal medicine cases. In this benchmark, performance differences between models were smaller in Phase 2 than in Phase 1. While diagnostic precision improves markedly, the models’ ability to generate comprehensive differential diagnoses that align with expert consensus is still limited. These findings underscore the utility of context-aware, multimodal LLMs for educational support and structured diagnostic practice in supervised settings while also highlighting the need for more sophisticated, semantics-sensitive benchmarks for evaluating diagnostic reasoning.

## 1. Introduction

Healthcare specialists rely mainly on visual inspection when diagnosing patients, predominantly in internal medicine contexts. These diagnostic results often originate from what doctors observe in various imaging techniques which may include skin presentations, X-rays, computed tomography scans, electrocardiogram readings, and microscopic tissue samples. Specialists highlight how lesion characteristics and their distribution guide dermatological diagnosis, while also stressing the need to create visual data across different medical specialties into rational diagnostic assessments [1,2]. Diagnosis is not only a visual exercise; research demonstrates that even brief patient histories can maximize the accuracy of image interpretation, revealing how visual evidence and narrative details work together in clinical decision making [3]. This reality pushes interest in large language models as tools for both education and clinical decision support. Initial research indicates these models can handle medical questions and standardized examinations, though their dependability appears linked to input characteristics [4].

Initial assessments put general-purpose large language models as capable players in text-based medical challenges. ChatGPT achieved passing or near-passing scores on United States Medical Licensing Examination (USMLE) assessments and generated clinically rigorous explanations, directing toward utility in educational settings [4]. Follow-up experimental work using standardized clinical scenarios demonstrated that these models can organize differential diagnoses and explain their reasoning, though both accuracy and explanation quality fluctuate based on case difficulty and how questions are framed [5]. Direct model comparisons using specialty examination databases showed mixed results, revealing how sensitive outcomes are to subject matter coverage and instruction phrasing [6]. Complementing these single-study findings, recent reviews synthesize a fast-moving literature and converge on a theme: LLMs exhibit promise for medical decision support and education, yet evidence remains uneven across tasks and modalities. Limitations noted in this body of work include sparse assessment of visual, image-first problems and inconsistent treatment of differential diagnosis scoring or the incremental effect of adding brief clinical history [7].

Recent work has begun to test multimodal large models on real medical images across specialties. In radiology, several groups evaluated GPT-4V or related vision-language models on chest radiographs and mixed-modality studies, showing that these systems can extract key findings and answer image-grounded questions, albeit with variable reliability across tasks and datasets [8,9]. Broader assessments using curated radiology case collections similarly reported that GPT-4V outperforms text-only LLM baselines when image input is available, emphasizing the potential value of visual context in diagnostic reasoning [10]. Outside radiology, dermatology studies indicate that multimodal models can separate benign from malignant lesions and generate clinically sensible descriptions, suggesting applicability to pattern-recognition problems at the bedside [11]. In pathology, emerging domain-specific artificial intelligence (AI) and early ChatGPT-based evaluations on microscopic images illustrate growing feasibility of image-aware reasoning in morphologic diagnosis, though methods and benchmarks remain heterogeneous [12,13]. Parallel systematic reviews examining medical artificial intelligence systems have captured these patterns, finding that vision-language models show potential for report creation and visual question answering tasks. However, these reviews also underscore the necessity for uniform evaluation methods across various clinical fields [14,15,16].

Despite artificial intelligence’s remarkable advances in processing diverse medical information, surprisingly few investigations have tested its capability on simple, single-image scenarios in general internal medicine. Such studies seldom employ tightly controlled experimental designs using just two conditions to definitively measure how much a brief patient narrative actually improves performance. Equally rare are investigations that assess how well artificial intelligence-generated differential diagnoses correspond to expert consensus while simultaneously measuring overall diagnostic precision. Recent systematic reviews have called for developing standardized assessment tools spanning multiple medical disciplines with explicit scoring frameworks. These benchmarks should extend beyond purely text-based case presentations and explicitly demonstrate how supplementary context modifies outcomes, building on emerging work on rigorous imaging-AI evaluation and attention-driven analysis [17,18]. This research gap stands out given long-established evidence that providing concise clinical histories can alter image interpretation and reshape the relevance of proposed differential diagnoses [19]. Harrison’s Visual Case Challenge offers expert-selected, image-centered cases complete with authoritative diagnoses and reference differential lists—materials that reflect how internists practice visual pattern recognition and how medical students are evaluated—thereby providing a standardized, reproducible benchmark for testing whether brief clinical context enhances artificial intelligence diagnostic performance [20]. This investigation sought to directly compare ChatGPT, Gemini, and DeepSeek on 138 Harrison’s cases. Our primary objective was to determine whether adding a brief clinical history improves top-1 diagnostic accuracy of general-purpose multimodal LLMs on visual internal medicine cases. Using this single endpoint, we compared three models (ChatGPT, Gemini, and DeepSeek). Secondary/Exploratory analyses assessed (i) the magnitude of the history-associated change in accuracy and (ii) agreement between model-generated differential lists and the textbook differential.

## 2. Method

### 2.1. Study Design

We conducted a comparative evaluation of three large language models—ChatGPT, Gemini, and DeepSeek—using image-based clinical cases from Harrison’s Visual Case Challenge under a standardized evaluation workflow [20]. Prompt wording, decoding parameters, and case order were held constant across models.

### 2.2. Phases and Prompting

In Phase 1 (image only), the case image—downloaded directly from Harrison’s Visual Case Challenge on AccessMedicine (McGraw Hill)—was uploaded to each model using its native image attachment interface, with no accompanying clinical text; the prompt asked for a single best diagnosis and instructed the model not to request additional information. Immediately after recording the Phase 1 output for the same case, we proceeded to Phase 2 (image + brief history). The identical image was presented again together with the concise patient history as written in the book, and the prompt requested a single best diagnosis plus a brief differential diagnosis list. The Phase 2 histories were used verbatim and were not edited or standardized; their length and included elements vary by case (e.g., demographics, symptom timing, and occasional laboratory clues), consistent with the textbook format. Prompts were standardized across models, decoding parameters were held constant, and no external tools or web browsing were permitted. The study intentionally used a minimal, fixed prompt and did not introduce additional prompt variants (e.g., explicit reasoning instructions or top-k answer formats) in this benchmark.

### 2.3. Data Source and Case Selection

Cases were drawn from *Harrison’s Visual Case Challenge* (McGraw-Hill; AccessMedicine). All 138 cases with a single representative image and a definitive reference diagnosis were included. Because the corpus contains a fixed set of 138 cases (*n* = 138), no formal a priori power calculation was performed; however, this sample size allows reasonably precise estimation of accuracy (95% confidence intervals of roughly ±8 percentage points around proportions near 0.5) and provides adequate power to detect moderate differences between phases and between models. These challenge cases are educational, single-image vignettes curated for teaching and are not prevalence-weighted, emphasizing classic “textbook” presentations rather than real-world incidence. Cases with a provided expert differential list were flagged for the differential-agreement analyses. For each case we recorded the clinical system/domain, a disease nature category (e.g., inflammatory/autoimmune, infectious, neoplastic), and the image modality (e.g., dermatology photograph, radiograph/CT, ECG, pathology). No cases contained patient identifiers. Because Harrison’s content is widely used in medical education, training data contamination cannot be excluded, and we did not attempt to distinguish cases that might be familiar to the models from those that are likely novel.

### 2.4. Reference Answer

For each case, the book’s single correct diagnosis served as the reference. A reference differential diagnosis list is provided for all cases and was used to assess differential agreement. Primary diagnosis scoring followed the book’s term, with limited manual synonym allowance at the author’s discretion (no formal ontology or automated normalization). Differential agreement was summarized as percent overlap, i.e., matched terms divided by the number of terms in the book’s list.

### 2.5. Models and Configurations

ChatGPT (version: GPT-5 default), Gemini (version: Gemini 2.5 Flash), and DeepSeek (version: DeepSeek-V3.2) were accessed via their websites on the free (unpaid) tier between 1 and 30 September 2025. Model/version identifiers were recorded exactly as displayed in each platform’s model selector during the evaluation window. All interactions used default settings; no plug-ins, retrieval, or web browsing were enabled, and identical prompts were used across models. Thus, the results represent a time-bounded snapshot of each platform under free-tier constraints, which may include undocumented limits on context length, response length, or image resolution and could affect absolute performance.

### 2.6. Outcomes

The primary outcome was top-1 diagnostic accuracy per case for each model in Phase 1 (image only, without any patient history) and Phase 2 (image plus the book’s brief patient history). Top-1 accuracy is reported as a benchmark endpoint under the study’s single diagnosis prompt constraint and should not be interpreted as a direct clinical top-k usefulness measure. To ensure reproducible scoring, we normalized diagnosis labels using a pre-specified equivalence list covering clinically trivial wording variants (including unambiguous abbreviations and established eponyms). Before analysis, we created this synonym list for all distinct diagnosis labels; two clinician authors curated it with SNOMED CT used as a reference where needed, and disagreements were resolved by a third author. Diagnoses were scored correct only when they matched the normalized concept label exactly (partial string matches were not credited), and umbrella diagnoses were not credited unless identical to the book’s reference diagnosis concept. The single diagnosis was counted as correct when it exactly matched the book’s single correct diagnosis. For example, ‘MI’ was treated as equivalent to ‘myocardial infarction,’ whereas broader related terms such as ‘acute coronary syndrome’ were not counted as a match unless identical to the reference diagnosis concept. The secondary outcome was differential diagnosis agreement, defined as the percentage of diagnoses in the book’s reference differential list that also appeared in the model’s differential diagnosis list (matched terms ÷ total terms in the book’s list × 100). We summarized Phase 2 differential performance using three set-based metrics: (i) differential coverage, defined as the proportion of cases in which at least one textbook differential term appeared in the model’s differential list (case-level hit rate); (ii) recall of the textbook differential list, defined as matched textbook terms ÷ total textbook differential terms; and (iii) Jaccard overlap, defined as matched terms ÷ the union of textbook and model differential term sets. Differential lists were evaluated as unordered sets (list order ignored), and rank-aware metrics were therefore not applied. For example, ‘MI’ was treated as equivalent to ‘myocardial infarction,’ whereas broader related terms such as ‘acute coronary syndrome’ were not counted as a match unless identical to the reference diagnosis concept.

### 2.7. Statistical Analysis

For each model and analysis stratum we reported the proportion accuracy with Wils on 95% confidence intervals. Paired comparisons of accuracy used McNemar’s test (within case), including (i) model-versus-model contrasts within a phase and (ii) Phase 1 versus Phase 2 within model. For paired contrasts (Phase 1 vs. Phase 2 within model; and model-versus-model within phase), we additionally report the absolute difference in accuracy with 95% CIs computed using a paired-proportion CI method based on discordant pairs. When comparing all three models simultaneously, we used Cochran’s Q with post hoc McNemar tests and Benjamini–Hochberg correction for multiple testing. For differential diagnosis agreement, we reported the case-level percent overlap as mean (SD) and median [IQR]; paired between-model comparisons used nonparametric tests (e.g., Wilcoxon signed-rank) where appropriate. Analyses were performed using Python (version 3.14.0). Two-sided *p* < 0.05 was considered statistically significant after correction where applicable.

### 2.8. Ethics and Copyright

All cases were drawn from Harrison’s Visual Case Challenge and contained no identifiable patient information. Representative case images and verbatim histories cannot be reproduced in the manuscript due to publisher copyright. This project involved no human–subjects interaction; an institutional review board (IRB) review was therefore not required or was deemed exempt under local policy. To support reproducibility, the complete prompt templates and platform configurations, scoring procedures, and case index with case-set IDs and A/B image labels, together with an outputs template, are provided in Supplementary Files S1 and S2.

## 3. Results

### 3.1. Overall Diagnostic Accuracy

We analyzed 138 cases from Harrison’s Visual Case Challenge to estimate the top-1 diagnostic accuracy of ChatGPT, Gemini, and DeepSeek and to compare performance across the two phases. Table 1 summarizes overall counts and per-phase accuracies for each model. Adding a brief clinical history improved performance for all three models. In Phase 1 (image only), accuracies were 50.72% for ChatGPT (70/138), 39.90% for Gemini (55/138), and 30.43% for DeepSeek (42/138). In Phase 2 (image + brief history), accuracies rose to 80.40% for ChatGPT (111/138), 72.50% for Gemini (100/138), and 75.36% for DeepSeek (104/138). The corresponding absolute improvements were +29.70 percentage points (pp) for ChatGPT, +32.60 pp for Gemini, and +44.93 pp for DeepSeek, indicating that concise clinical context was associated with sizable gains across models. As a contamination sensitivity analysis, after excluding dermatology and ocular cases (axes 1–3; 50/138 images), the main conclusion was unchanged and diagnostic accuracy still improved from Phase 1 to Phase 2 for all models (ChatGPT 55.7% vs. 79.5%; Gemini 20.5% vs. 67.0%; DeepSeek 36.4% vs. 77.3%).

### 3.2. Performance by Disease Nature Category

Table 2 presents diagnostic performance stratified by disease nature category, with Phase 1 and Phase 2 accuracies and changes for each model. Key findings include consistent improvements from Phase 1 to Phase 2 for ChatGPT and DeepSeek across all categories, while Gemini showed mixed results with gains in most but declines in two (metabolic/toxic: −10.00 pp; arrhythmic/electrophysiological: −20.00 pp). DeepSeek demonstrated the most substantial gains overall (e.g., +75.00 percentage points [pp] in cutaneous inflammatory/autoimmune and viral/parasitic infections), though its Phase 1 performance was very poor. In general, Phase 1 accuracies varied widely, with Gemini performing best (highest in 7 of 10 categories, e.g., 90.00% in structural/degenerative, 85.00% in arrhythmic/electrophysiological) and DeepSeek worst (lowest in 9 of 10 categories, e.g., 0.00% in viral/parasitic infections, 5.00% in arrhythmic/electrophysiological); ChatGPT was intermediate (e.g., 100.00% in “others” but 20.00% in metabolic/toxic). In Phase 2, accuracies converged to ≥65.00% in most categories, with DeepSeek leading or tying in four categories (e.g., 85.00% in cutaneous inflammatory/autoimmune, 80.00% in metabolic/toxic), ChatGPT in five categories (e.g., 100.00% in structural/degenerative and traumatic/hemorrhagic), and Gemini in three categories.

**Figure 1:**
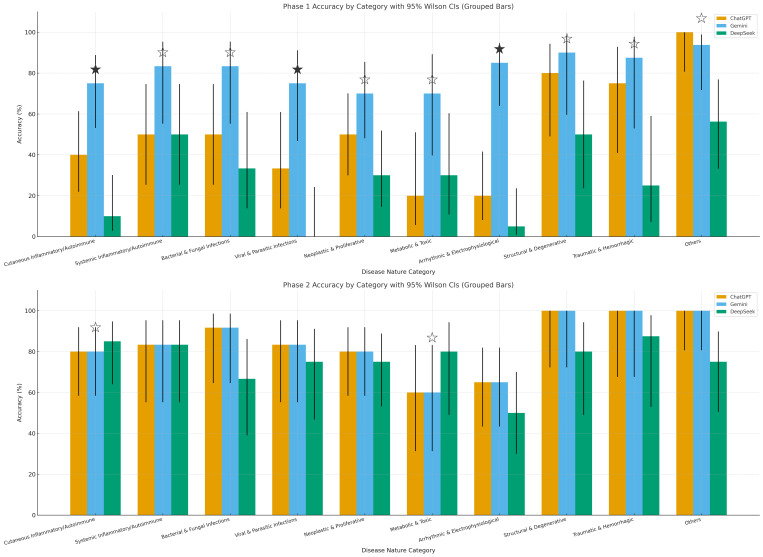
Phase-wise accuracy by category with 95% CIs (grouped bars). Phase-wise (Phase 1 **top**; Phase 2 **bottom**) per-category diagnostic accuracy for ChatGPT, Gemini, and DeepSeek on 138 images across 10 disease nature categories. Bars show % correct with Wilson 95% CIs. A solid star (★) marks a single highest model whose lead over the next best is statistically significant (Newcombe 95% CI for the difference excludes 0). A white/hollow star (☆) indicates the model is highest but not significant; ties have no star.

Figure 1 visualizes these accuracies as grouped bars with 95% Wilson confidence intervals (CIs; Phase 1 on top, Phase 2 on bottom), highlighting inter-model comparisons with statistical markers. Important findings include broader CIs in smaller categories (e.g., traumatic/hemorrhagic, n = 8), indicating greater variability, and more distinct bar separations in Phase 1 than Phase 2. In Phase 1, Gemini often led with significant superiority (solid stars) in categories like arrhythmic/electrophysiological and cutaneous inflammatory/autoimmune (Newcombe’s 95% CI for differences excludes 0; *p* < 0.05), while DeepSeek rarely competed. In Phase 2, differences were less marked, suggesting that added history can help reduced disparities.

**Figure 2:**
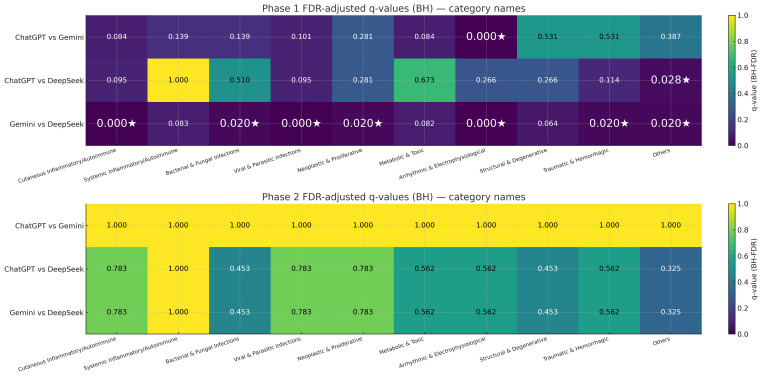
Pairwise significance after multiple-testing correction (FDR heatmaps). False discovery rate (FDR)-adjusted q-values (Benjamini–Hochberg) for pairwise model comparisons across categories in Phase 1 (**top**) and Phase 2 (**bottom**). Rows correspond to ChatGPT vs. Gemini, ChatGPT vs. DeepSeek, and Gemini vs. DeepSeek; columns are the 10 categories. Cell values are q-values (darker = smaller), with a white star (★) marking q < 0.05. This panel answers where model differences are statistically credible after controlling for multiplicity.

Figure 2 presents false discovery rate adjusted q-values (Benjamini–Hochberg) as heatmaps comparing the models pairwise, where darker shades with lower q-values closer to zero point to stronger significance in a friendly, easy-to-spot way. The findings indicate greater differences in Phase 1. Q-values below 0.05 were frequently observed for Gemini versus DeepSeek, notably q = 0.000 in arrhythmic/electrophysiological, cutaneous inflammatory/autoimmune, and neoplastic/proliferative cases. Some differences were also noted for ChatGPT against DeepSeek, with a q-value of 0.028 in the “others” category. In contrast, ChatGPT versus Gemini exhibited minimal significant results with most q-values exceeding 0.05. In Phase 2, q-values were consistently higher with no statistical significances, which demonstrates diminished inter-model variability after including clinical history and within this single textbook-derived benchmark, suggesting diminished inter-model variability after including clinical history.

### 3.3. Performance by Organ System Category

**Figure 3:**
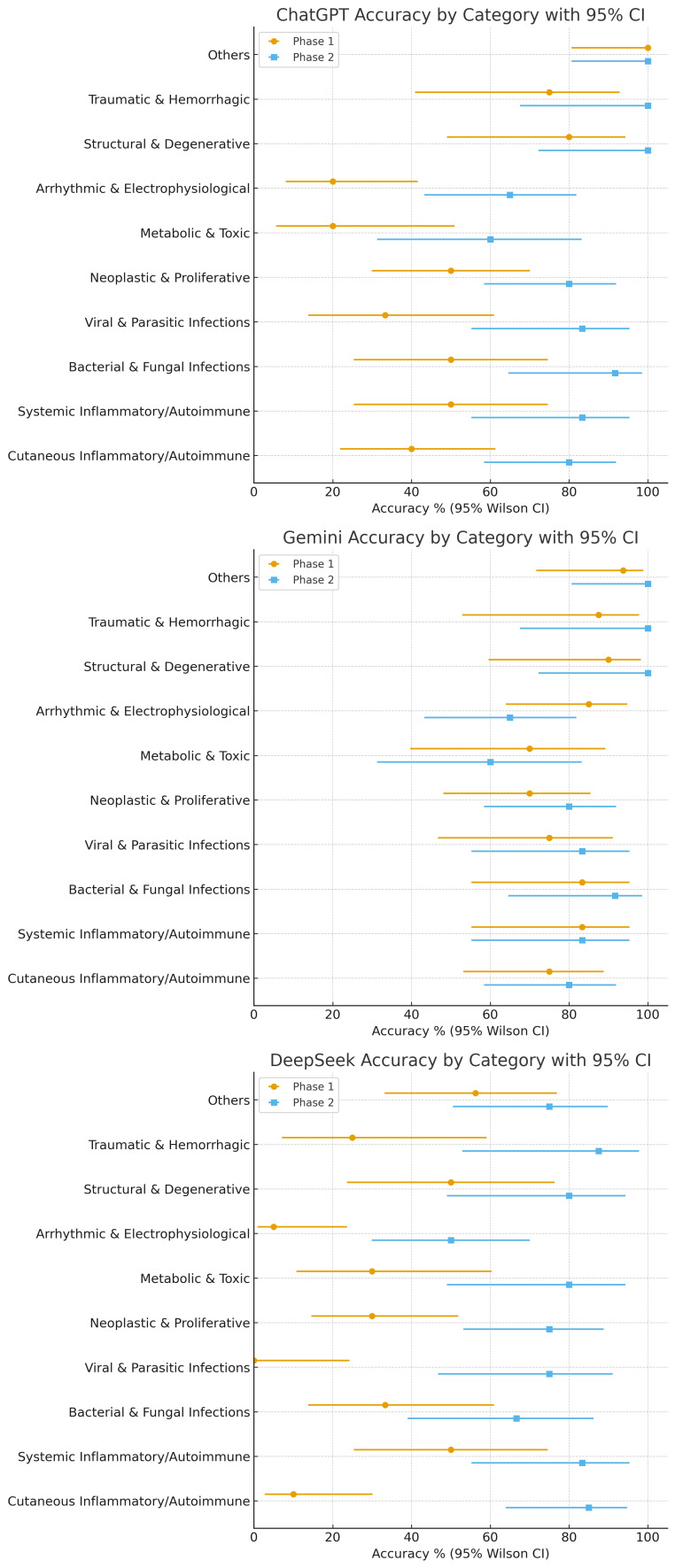
Model-centric dot-whisker plots with 95% CIs across categories.

Table 3 summarizes the diagnostic performance stratified by organ system category, presenting Phase 1 and Phase 2 accuracies along with changes for each model. The accuracies varied widely among AI models in Phase 1. ChatGPT performed best in pulmonary/thoracic (100%) and others (100%) category cases. Gemini outperformed in cardiovascular (83.3%) and neurological (91.7%) systems cases, while DeepSeek lagged significantly with the lowest accuracies in ocular (0%) and oral/mucosal (0%) systems cases. In Phase 2, all three AI models showed significant improvement, achieving ≥75.00% in most categories. The most significant improvements were for ChatGPT’s performance (+57.10 pp) in hematological/fluid systems (from 28.60% to 85.70%), DeepSeek’s +100.00 pp in oral/mucosal (from 0% to 100%), and consistent high performance in pulmonary/thoracic (100.00% for ChatGPT and Gemini, 75.00% for DeepSeek). The dot-whisker plots highlighting these shifts are presented in Figure 3 with tighter CIs in larger categories (e.g., blistering/nodular skin disorders, n = 28) and broader CIs in smaller ones (e.g., pulmonary/thoracic, n = 4), reflecting sample size effects.

Per-model accuracy by category with 95% Wilson Cis are shown as dot-whisker plots for (left-to-right) ChatGPT, Gemini, and DeepSeek. For each category, markers display Phase 1 and Phase 2 side-by-side, enabling a direct visual of within-model improvements and remaining variability across categories.

### 3.4. Differential Diagnosis Precision

**Figure 4:**
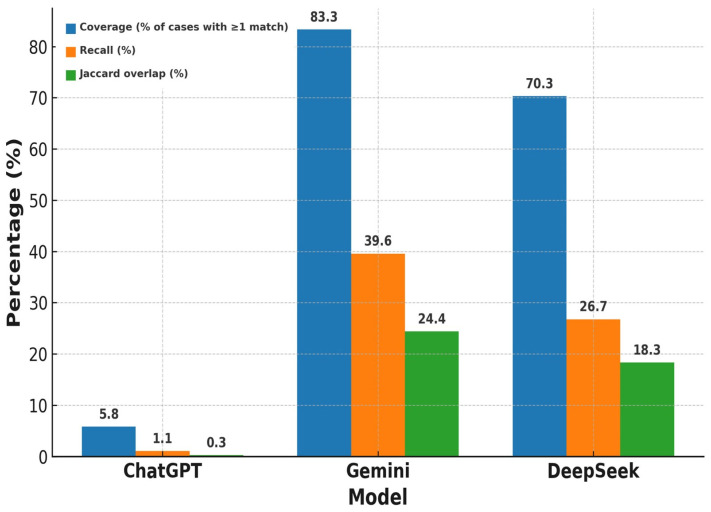
Phase 2 differential diagnosis coverage, recall, and Jaccard overlap by model.

For Phase 2 only, differential diagnosis precision (percentage overlap with differential diagnosis mentioned in the book) is summarized in Table 4 with categorization as per organ system. The precision varied widely across all three AI models in general. ChatGPT averaged 6.99% (median 6.47%; range 0.0–21.42%), significantly lower than Gemini at 36.39% (median 37.5%; range 0.0–92.85%) and DeepSeek at 32.74% (median 33.33%; range 0.0–87.5%). This divergence from ChatGPT’s high Phase 2 top-1 accuracy reflects that top-1 scoring applied clinician-curated normalization for trivial label variants, whereas differential agreement was evaluated as strict term overlap against the textbook list (order ignored), which is more sensitive to lexical choices and brief lists. Gemini and DeepSeek consistently outperformed ChatGPT in some categories. In microscopy (urine/synovial fluid) cases, Gemini achieved 45% and DeepSeek achieved 50%, while ChatGPT scored just 7.74%. For papulosquamous skin photos, Gemini scored 43.75% and DeepSeek 37.49%, compared with ChatGPT’s 10.49%. The ranges showed differences with Gemini peaking at 92.85% in ocular photographs and DeepSeek at 87.5% in papulosquamous skin photos, while ChatGPT topped out at 21.42% in ECG cases. The set-based summaries of the Phase 2 differential diagnoses are shown in Figure 4. Under our matching criteria, at least one textbook differential term appeared in only 5.8% of cases for ChatGPT (8/138), but in 83.3% and 70.3% of cases for Gemini and DeepSeek, respectively. Mean recall of the textbook differential list was 1.1% for ChatGPT, 39.6% for Gemini, and 26.7% for DeepSeek, with corresponding mean Jaccard overlaps of 0.26%, 24.4%, and 18.3%, respectively.

## 4. Discussion

Evaluating ChatGPT, Gemini, and DeepSeek on 138 cases from Harrison’s Visual Case Challenge showed that adding brief clinical histories dramatically boosted diagnostic performance. DeepSeek showed the most striking gains, jumping from a modest Phase 1 baseline of 30.43% accuracy to 75.36%—a remarkable increase of 44.93 percentage points. Gemini AI led competitors in Phase 1 visual diagnostics, excelling at pure image interpretation across comparable benchmarks, which underscores its multimodal pattern recognition capabilities even without contextual support. When context was added in Phase 2, accuracies across models became more similar, as reflected in elevated q-values, suggesting diminished between-model variation. That said, generating precise differential diagnoses proved difficult for all models. ChatGPT averaged merely 6.99% alignment (ranging from 0.0% to 21.42%), while Gemini achieved 36.39% (0.0% to 92.85%) and DeepSeek reached 32.74% (0.0% to 87.5%). These figures reveal ongoing struggles to produce thorough differential lists, despite some categories like ocular photographs showing notably higher ranges. The results point to large language models as promising supplementary tools when clinical history is available, yet they continue to face obstacles in pure image interpretation, especially for complex presentations. Given this modest differential alignment, we interpret the current findings primarily as supporting educational use (e.g., supervised teaching and structured practice) rather than stand-alone diagnostic assistance [21,22].

At a more granular level, the stratified analyses by disease nature category, organ system, and imaging modality show that the general pattern of Phase 2 improvement holds across most internal medicine domains, with some variation in the magnitude of gains by model and category. Qualitatively, many of the remaining errors involve visually or clinically similar entities (e.g., look-alike dermatoses or rhythm-strip patterns) rather than completely unrelated diagnoses, which may be helpful when using these outputs in teaching and case discussion. By modality, the most common failure modes differed. In dermatology photographs, incorrect top-1 outputs most often reflected visually similar mimics and near-miss look-alike dermatoses. In ECG or rhythm-strip cases, errors often reflected confusion between similar rhythm patterns. In CT or MRI cases, misses were more common when findings were subtle or nonspecific on a single representative image, and brief history could help narrow interpretation.

Our Phase 1 to Phase 2 gains (spanning +29.70 to +44.93 percentage points) echo Han et al. (2023)’s observations that multimodal large language models surpass baseline performance when provided clinical context [23]. Hu et al. (2023) reported that LLMs enhance pathology tasks with contextual input, specifically highlighting the performance of models like GPT-3 and BERT in improving diagnostic accuracy when paired with clinical context in pathology image analysis [24]. Bradshaw et al. (2025) also observed that adding a patient’s background or brief history greatly improves the accuracy of multimodal models in correctly assessing cardiac images [25]. In the current study, 138 cases spanning dermatology photographs, ECG tracings, chest X-rays, and CT/MRI scans offer a comprehensive view of internal medicine diagnostics moving beyond single-modality tests. Clear reporting of evaluation design and scoring procedures is emphasized across medical imaging AI studies [26]. Phase 1 accuracies ranged from 30 to 50%, but adding brief patient history like age, symptoms, and labs boosted Phase 2 accuracies to 80.43% for ChatGPT (+29.7 pp), 72.46% for Gemini (+32.60 pp), and 75.36% for DeepSeek (+44.93 pp). For radiology-specific results, cardiovascular cases (n = 24) improved from 4.17% to 54.17% for DeepSeek (+50 pp) and from 25.00% to 62.50% for ChatGPT (+37.5 pp), though Gemini dropped from 83.30% to 62.50% (−20.80 pp), while neurological CT/MRI brain scans (n = 12) rose from 33.33% to 91.67% for DeepSeek (+58.34 pp), showing history’s role in refining subtle findings. This approach aligns with Suh and Shim (2024), who reported top-1 accuracy on 190 radiology cases jumping from 15% (image-only) to 48% with patient history images [27], and the Kim et al. (2025), where Llama-3 and GPT-4o achieved 70–80% accuracy on 1933 cases with full history, emphasizing context’s impact—though our mixed internal medicine focus and pure-image Phase 1 baseline offer a broader contrast than their subspecialty baselines [28]. Related vision-language model benchmarking in radiology has also evaluated multimodal models on multisequence MRI datasets using structured comparisons [29].

Gemini displayed strong Phase 1 capabilities, hitting 85% accuracy for arrhythmic and electrophysiological cases—a finding that resonates with Kao et al. (2025)’s work on large language model effectiveness in radiology pattern recognition [30] and Bradshaw et al. (2024)’s validation of deep learning for computed tomography analysis, both pointing to solid initial visual interpretation skills [25]. By contrast, our study found ChatGPT delivered steady incremental improvements, including a noteworthy +57.10 percentage point gain in hematological and fluid system cases, which mirrors Han et al. (2024)’s comparative assessment of text-based reasoning and suggests balanced flexibility across different contextual scenarios [23]. DeepSeek’s context-driven improvement stands out, particularly its +75.00 percentage point rise in cutaneous cases, which aligns with Ling et al. (2023)’s focus on domain specialization [31] and mirrors the well-documented constraints of multimodal AI in clinical settings, such as data harmonization and model integration [32]. However, its weak Phase 1 showing (including 0.00% in viral cases) diverges from the consistent visual competence reported by Qin et al. (2023), highlighting its reliance on contextual information [33]. Gemini’s −20.00 percentage point drop in arrhythmic cases contrasts with the stable reporting seen in Atsukawa et al. (2025)’s radiology research [34] and Hu et al. (2023)’s imaging advances [24], likely reflecting the complexity of arrhythmic visuals. Worth noting, Suh & Shim (2024) documented similar initial strength in Gemini Pro Vision that weakened without historical context [27], contrasting with the Eurorad Benchmark (2025)’s Llama-3, which maintained stability when given contextual support [28]. Our comparative analysis reveals this variability, a dimension often overlooked in single-model investigations such as Bradshaw et al. (2024) [25] and Sahoo et al. (2024), prompt engineering study [35], thereby extending comprehensive evaluation by identifying model-specific deficiencies in visual task performance [33,36].

Differential diagnosis accuracy (Phase 2) varied among AI models, i.e., Gemini 36.39%, DeepSeek 32.74%, and ChatGPT 6.99% overall with wide spread across imaging categories. Accuracy peaked in stereotyped visuals—ocular photographs up to 92.85% and papulosquamous dermatoses up to 87.5%, but it was lower for ECG/rhythm (ChatGPT’s maximum: 21.42%). Because overlap reflects precision rather than completeness, future work should add recall/Jaccard or concept-level metrics to capture missed but clinically relevant alternatives. In a comparison of two LLMs with a legacy diagnostic decision support system on 36 unpublished cases, the share of cases where the correct diagnosis appeared anywhere in a 25-item differential was 42% (LLM1) and 39% (LLM2) without labs, rising to 64% and 58% with labs [37]; the DDSS listed the diagnosis more often and higher than the LLMs. Our differential precision numbers (Gemini 36.39%, DeepSeek 32.74%) are broadly consistent with that mid-range inclusion band once limited context is provided, though we score by term overlap rather than presence/rank, which is a stricter criterion.

Domain-specific evaluations echo this. In dermatology, a vision-enabled LLM correctly identified the top diagnosis in 54% of image-only cases and included the correct diagnosis in the differential in 50%, highlighting that differentials can capture partial clinical alignment even when the single best guess is wrong—much like our category-level peaks in ocular and papulosquamous groups [38]. In multimodal general clinical testbeds, investigators have likewise emphasized top-k differential metrics as more informative than top-1 alone; adopting such multi-term measures would complement our precision overlap and likely raise measured agreement for cases where models named several correct alternatives [39].

A key strength of the current study is the standardized, expert-curated corpus from *Harrison’s Visual Case Challenge* with canonical answers and reference differentials, enabling transparent, reproducible comparisons across models and conditions. The two-condition design (image-only vs. image + history) directly quantifies the incremental contribution of brief context at scale (n = 138). Limitations include reliance on a single educational, prevalence-agnostic textbook source and single-image vignettes (real-world cases may involve multi-view or serial imaging), which may introduce spectrum bias by over-representing classic presentations and under-representing borderline, ambiguous, or comorbid cases; real-world imaging may also be noisy or incomplete. We also did not obtain clinician performance on the same images under identical constraints, so we cannot directly compare LLM accuracy with expert or trainee benchmarks. In addition, we evaluated free-tier models during a fixed window (September 2025) under platform-specific constraints that may limit context length, throughput, or image resolution. We did not repeat the full experiment across multiple days, so our findings should be interpreted as a time-bounded snapshot that may change as model versions and free-tier policies evolve. Results reflect a single run per case and phase for each model; we did not repeat prompts to estimate within-model stochastic variability. While top-1 accuracy was pre-specified to match the study’s single diagnosis prompt and single reference label per case, future work can additionally assess top-3/top-5 inclusion using standardized ranked-list prompts. Model training corpora are not transparent, so training data contamination cannot be definitively excluded. We addressed this concern with a leave-out sensitivity analysis that excluded dermatology and ocular cases (axes 1–3). The Phase 2 improvement pattern was preserved across models. This supports the robustness of the main finding. The study did not quantify history information content (e.g., length or presence of age/labs/timing) or analyze accuracy gain as a function of these features, as Phase 2 histories were not standardized across cases. The study did not perform a prompt ablation (e.g., diagnosis-only vs. diagnosis + reasoning, or single diagnosis vs. top-k formats); therefore, some degree of prompt sensitivity cannot be excluded.

## 5. Conclusions

Across 138 single-image internal medicine cases, ChatGPT, Gemini, and DeepSeek achieved moderate to high top-1 diagnostic accuracy, with performance varying by disease category and modality. Providing a brief clinical history further improved accuracy for all three models and reduced apparent performance gaps between models within this benchmark. While variations across categories persisted, diagnostic accuracy generally aligned once clinical context was provided. Correspondence with expert differential diagnoses stayed moderate overall, with stronger agreement in visually characteristic modalities and weaker matching in rhythm interpretation. These outcomes support using multimodal models with clinical history for educational support and structured practice in supervised settings, while highlighting the ongoing need for standardized, semantically informed assessment of differential diagnosis lists. Future work should extend beyond a single educational source and single-image cases, incorporate ontology-anchored scoring for differentials, and include prospective comparisons with clinicians in blinded, multi-center settings.

## Figures and Tables

**Table 1:** Overall diagnosis accuracy and differential diagnosis precision for ChatGPT, Gemini, and DeepSeek in medical image diagnosis.

Metric	CHATGPT			GEMINI			DEEPSEEK		
	Phase 1 N (%)	Phase 2 N (%)	Δ Difference	Phase 1 N (%)	Phase 2 N (%)	Δ Difference	Phase 1 N (%)	Phase 2 N (%)	Δ Difference
	**Diagnosis Accuracy**								
Total Cases	138	138	—	138	138	—	138	138	—
Correct Diagnoses	70 (50.72%)	111 (80.43%)	+41 (+58.57%)	55 (39.86%)	100 (72.46%)	+45 (+81.82%)	42 (30.43%)	104 (75.36%)	+62 (+147.62%)
Incorrect Diagnoses	68 (49.28%)	27 (19.57%)	−41 (−60.29%)	83 (60.14%)	38 (27.54%)	−45 (−54.22%)	96 (69.57%)	34 (24.64%)	−62 (−64.58%)
Overall Accuracy	50.70%	80.40%	+29.70 ppt	39.90%	72.50%	+32.60 ppt	30.43%	75.36%	+44.93 ppt
	**Differential Diagnosis (Phase 2 Only) Accuracy**								
Average (%)	6.99%			36.39			32.74		
Range (Min %–Max %)	0.0–21.42			0.0–92.85			0.0–87.5		
Median %	6.47			37.5			33.33		

**Table 2:** Comparative performance of ChatGPT, Gemini, and DeepSeek in medical image diagnosis across disease nature categories: correct diagnoses and accuracy changes between Phase 1 and Phase 2 for 138 cases.

Disease Nature Category	Total Cases	CHATGPT			GEMINI			DEEPSEEK		
		Phase 1 Correct (% Acc.) Diagnosis	Phase 2 Correct (% Acc.) Diagnosis	Difference Δ % Accuracy	Phase 1 Correct (% Acc.) Diagnosis	Phase 2 Correct (% Acc.) Diagnosis	Difference Δ % Accuracy	Phase 1 Correct (% Acc.) Diagnosis	Phase 2 Correct (% Acc.) Diagnosis	Difference Δ % Accuracy
Cutaneous Inflammatory/Autoimmune	20	8 (40.00%)	16 (80.00%)	+40.00 ppt	15 (75.00%)	16 (80.00%)	+5.00 ppt	2 (10.00%)	17 (85.00%)	+75.00 ppt
Systemic Inflammatory/Autoimmune	12	6 (50.00%)	10 (83.30%)	+33.30 ppt	10 (83.30%)	10 (83.30%)	0.00 ppt	6 (50.00%)	10 (83.33%)	+33.33 ppt
Bacterial and Fungal Infections	12	6 (50.00%)	11 (91.70%)	+41.70 ppt	10 (83.30%)	11 (91.70%)	+8.40 ppt	4 (33.33%)	8 (66.67%)	+33.34 ppt
Viral and Parasitic Infections	12	4 (33.30%)	10 (83.30%)	+50.00 ppt	9 (75.00%)	10 (83.30%)	+8.30 ppt	0 (0.00%)	9 (75.00%)	+75.00 ppt
Neoplastic and Proliferative	20	10 (50.00%)	16 (80.00%)	+30.00 ppt	14 (70.00%)	16 (80.00%)	+10.00 ppt	6 (30.00%)	15 (75.00%)	+45.00 ppt
Metabolic and Toxic	10	2 (20.00%)	6 (60.00%)	+40.00 ppt	7 (70.00%)	6 (60.00%)	−10.00 ppt	3 (30.00%)	8 (80.00%)	+50.00 ppt
Arrhythmic and Electrophysiological	20	4 (20.00%)	13 (65.00%)	+45.00 ppt	17 (85.00%)	13 (65.00%)	−20.00 ppt	1 (5.00%)	10 (50.00%)	+45.00 ppt
Structural and Degenerative	10	8 (80.00%)	10 (100.00%)	+20.00 ppt	9 (90.00%)	10 (100.00%)	+10.00 ppt	5 (50.00%)	8 (80.00%)	+30.00 ppt
Traumatic and Hemorrhagic	8	6 (75.00%)	8 (100.00%)	+25.00 ppt	7 (87.50%)	8 (100.00%)	+12.50 ppt	2 (25.00%)	7 (87.50%)	+62.50 ppt
Others	16	16 (100.00%)	16 (100.00%)	0.00 ppt	15 (93.75%)	16 (100.00%)	+6.25 ppt	9 (56.25%)	12 (75.00%)	+18.75 ppt

**Table 3:** Comparative performance of ChatGPT, Gemini, and DeepSeek in medical image diagnosis across organ systems.

Organ System Category	Total Cases	CHATGPT			GEMINI			DEEPSEEK		
		Phase 1 Correct (% Acc.) Diagnosis	Phase 2 Correct (% Acc.) Diagnosis	Difference Δ % Accuracy	Phase 1 Correct (% Acc.) Diagnosis	Phase 2 Correct (% Acc.) Diagnosis	Difference Δ % Accuracy	Phase 1 Correct (% Acc.) Diagnosis	Phase 2 Correct (% Acc.) Diagnosis	Difference Δ % Accuracy
Scaly Skin Disorders	12	4 (33.30%)	10 (83.30%)	+50.00 ppt	9 (75.00%)	10 (83.30%)	+8.30 ppt	2 (16.67%)	10 (83.33%)	+66.66 ppt
Blistering and Nodular Skin Disorders	28	12 (42.90%)	22 (78.60%)	+35.70 ppt	21 (75.00%)	22 (78.60%)	+3.60 ppt	8 (28.57%)	21 (75.00%)	+46.43 ppt
Ocular System	10	5 (50.00%)	9 (90.00%)	+40.00 ppt	7 (70.00%)	9 (90.00%)	+20.00 ppt	0 (0.00%)	5 (50.00%)	+50.00 ppt
Oral and Mucosal System	4	2 (50.00%)	4 (100.00%)	+50.00 ppt	3 (75.00%)	4 (100.00%)	+25.00 ppt	0 (0.00%)	4 (100.00%)	+100.00 ppt
Cardiovascular System	24	6 (25.00%)	15 (62.50%)	+37.50 ppt	20 (83.30%)	15 (62.50%)	−20.80 ppt	1 (4.17%)	13 (54.17%)	+50.00 ppt
Neurological System	12	9 (75.00%)	11 (91.70%)	+16.70 ppt	11 (91.70%)	11 (91.70%)	0.00 ppt	4 (33.33%)	11 (91.67%)	+58.34 ppt
Abdominopelvic and Gastrointestinal System	16	10 (62.50%)	14 (87.50%)	+25.00 ppt	13 (81.25%)	14 (87.50%)	+6.25 ppt	12 (75.00%)	13 (81.25%)	+6.25 ppt
Hematological and Fluid Systems	14	4 (28.60%)	12 (85.70%)	+57.10 ppt	11 (78.60%)	12 (85.70%)	+7.10 ppt	6 (42.86%)	13 (92.86%)	+50.00 ppt
Pulmonary and Thoracic System	4	4 (100.00%)	4 (100.00%)	0.00 ppt	4 (100.00%)	4 (100.00%)	0.00 ppt	2 (50.00%)	3 (75.00%)	+25.00 ppt
Others	14	14 (100.00%)	14 (100.00%)	0.00 ppt	13 (92.90%)	14 (100.00%)	+7.10 ppt	7 (50.00%)	11 (78.57%)	+28.57 ppt

**Table 4:** Comparative precision analysis of ChatGPT, Gemini, and DeepSeek in differential diagnosis across medical imaging categories: average, median, and range.

Category	Total Cases	CHATGPT Differential Diagnosis Accuracy			GEMINI Differential Diagnosis Accuracy			DEEPSEEK Differential Diagnosis Accuracy		
		Average % Precision	% Median	Range (Min–Max %)	Average % Precision	Median	Range	Average % Precision	Median	Range
Papulosquamous, Plaque, and Scaling Skin Photos	12	10.23	10.49	3.12–16.52	44.03	43.75	20.0–61.66	43.75	37.49	16.66–87.5
Vesicular, Ulcerative, Nodular, and Pigmented Skin Photos	28	5.63	5.52	0.0–11.46	35.45	32.08	10.0–77.5	36.01	39.58	0.0–66.66
Ocular and Conjunctival Photographs	8	4.64	0	0.0–18.58	37.8	29.17	0.0–92.85	32.29	20.83	12.5–75.0
Oral and Mucosal Photographs	4	7.6	7.6	6.54–8.66	30	30	10.0–50.0	41.67	41.67	0.0–83.34
Electrocardiography (ECG/Rhythm Strips)	24	9.01	10.04	0.0–21.43	39.14	43.75	7.14–87.5	23.75	16.66	0.0–62.5
Cross-Sectional Neuroimaging (CT/MRI Brain)	12	6.01	6.84	0.0–11.66	34.23	39.58	14.58–50.0	35.42	39.59	16.66–50.0
Cross-Sectional Neuroimaging (CT Abdomen/Pelvis)	12	6.78	5.32	0.0–18.34	36.9	39.64	12.14–55.0	27.64	26.66	0.0–58.34
Microscopy (Blood Smears and Parasites)	10	6.68	4.54	2.5–16.54	36.67	37.5	0.0–90.0	29.33	30	16.66–50.0
Microscopy (Urine and Synovial Fluid)	4	7.74	7.74	4.76–10.72	45	45	40.0–50.0	50	50	33.33–66.67
Others	24	6.2	6.08	0.0–14.36	30.28	33.33	12.5–62.5	30.68	33.33	12.5–50.0

## Data Availability

The datasets generated and/or analyzed during the current study are available from the corresponding author on reasonable request. The diagnostic images used in this study are drawn from *Harrison’s* *Visual Case Challenge* (McGraw-Hill; AccessMedicine) and are subject to publisher copyright; the raw images cannot be publicly shared by the authors. All non-image artifacts required to reproduce the evaluation—including full prompt templates and example wording, platform configuration and decoding settings, the case index with case-set IDs and A/B image labels, and an anonymized outputs template—are provided in Supplementary Files S1 and S2.

## Supplementary Materials

The following supporting information can be downloaded at: https://www.mdpi.com/article/10.3390/diagnostics16030388/s1.
