# Supplementary material for: A Comparative Assessment of ChatGPT, Gemini, and DeepSeek Accuracy: Examining Visual Medical Assessment in Internal Medicine Cases with and Without Clinical Context
Source: Diagnostics (Basel). 2026 Jan 26;16(3):388. doi: 10.3390/diagnostics16030388 (PMC12897363; doi:10.3390/diagnostics16030388)
Supplement: Supplementary file 1 [file diagnostics-16-00388-s001.zip › Supplementary File S1.pdf]

SUPPLEMENTARY FILE S1

Structure:

Section	Content Description
S1.1	Prompt Templates
S1.2	Example Interaction Format
S1.3	Platform Configurations and Decoding Settings
S1.4	Scoring Procedures
S1.5	Notes On Reproducibility

S1.1 PROMPT TEMPLATES

Phase 1 (image only, no history)

System / role instructions (generic): You are a board-certified internal medicine specialist. You will be shown a single diagnostic image from an internal-medicine case. Carefully examine the image and provide your single best working diagnosis. Do not invent clinical details beyond what is shown.

Example Phase 1 user prompt template: "You are given a single diagnostic image from an internal-medicine case.

[ATTACH IMAGE]

Task:
1. Carefully examine the image.
2. Provide ONE single best diagnosis.
3. Do NOT provide a differential diagnosis list.
4. Do NOT assume or invent any additional patient history or test results.
5. Answer in one line as: 'Final diagnosis: <diagnosis>'."

In the actual evaluation, the generic template above was instantiated for each case by attaching the textbook image through the model's native image-attachment interface. No clinical text was shown in Phase 1.

Phase 2 (image + brief history)

System / role instructions (generic): You are a board-certified internal medicine specialist. You will be shown a single diagnostic image and a brief patient history from an internal-medicine case. Carefully integrate the clinical history with the image to determine the most likely diagnosis and a short differential diagnosis list.

Example Phase 2 user prompt template: "You are given a single diagnostic image and a brief clinical history from an internal-medicine case.

[ATTACH IMAGE]

Patient history: Add brief history text copied verbatim from Harrison's Visual Case Challenge. Such as for Case Set 1- Image A: This patient is a 54-year-old man with a history of diabetes type II, who presents with severe left ear pain accompanied by drainage. The problem started 2 weeks ago after swimming in a lake. He had been ignoring it until he started noticing pain with chewing.

Task:
1. Carefully integrate the image and the clinical history.
2. Provide ONE single best diagnosis.
3. Then provide a short differential diagnosis list of other reasonable possibilities (2–4 items).
4. Do NOT assume or invent additional details beyond the history provided.

5. Answer in the following format:
   Final diagnosis: <diagnosis>
   Differential diagnosis: <diagnosis 1>; <diagnosis 2>; <diagnosis 3>; ..."

**Note: To reproduce the results with different AI models, prompt wording and task structure will be held constant across models and across all cases within each phase. No external tools or web browsing were permitted during the evaluation.*

S1.2 EXAMPLE INTERACTION FORMAT

For each case, the image was uploaded to the model via its native image-attachment interface. In Phase 1, only the image and the Phase-1 prompt template were provided. Immediately after recording the Phase-1 response for a given case, the same image was paired with the brief clinical history from the textbook and the Phase-2 prompt template was submitted for Phase 2. The order of cases and the Phase 1 → Phase 2 sequence were identical for all three models.

S1.3 PLATFORM CONFIGURATIONS AND DECODING SETTINGS

All models were accessed through their free, web-based interfaces. No plug-ins, retrieval, or browsing modes were enabled.

Model Configurations

- ChatGPT: platform-reported version label "GPT-5 default"; accessed via the free web interface between 1–30 September 2025.
- Gemini: platform-reported version label "Gemini 2.5 Flash"; accessed via the free web interface during the same period.
- DeepSeek: platform-reported version label "DeepSeek V3.0"; accessed via the free web interface during the same period.

Decoding and Interface settings

- Temperature: platform default (no explicit override).
- Top-p: platform default (no explicit override).
- Maximum tokens / response length: platform default (no explicit override).
- Additional tools: plug-ins, retrieval modes, and web browsing were disabled.
- For all platforms, identical prompts were used across models, and cases were presented in the same fixed order.

S1.4 SCORING PROCEDURES

Top-1 Diagnostic Accuracy

For each case, phase, and model, the final diagnosis string was extracted from the model's response. Top-1 diagnostic accuracy was defined as the proportion of cases for which the model's single final diagnosis exactly matched the textbook's single reference diagnosis for that case (term-level match after simple normalization of capitalization and punctuation).

Formally, for model m and phase p:

Accuracy(m, p) = (number of cases with an exact match between model diagnosis and reference diagnosis) / (total number of cases evaluated in that phase).

Differential-Diagnosis Agreement

For cases with a textbook differential-diagnosis list, we compared the model-generated differential list with the textbook list at the term level.

1. The textbook differential list was split into individual terms.
2. The model-generated differential list was split into individual terms using the same rules (semicolon- or comma-separated entries).
3. Terms were normalized (lowercasing and trimming whitespace).
4. We counted how many textbook differential terms also appeared anywhere in the model's list.

Differential agreement (percentage) was calculated as:

Differential agreement (%) = (number of textbook differential terms present in the model's differential list) / (total number of textbook differential terms) × 100.

This scoring focuses on the textbook's reference differential list and does not penalize additional extra terms suggested by the model.

Ontology mapping

For some analyses, both reference diagnoses and model predictions can be mapped to standard concepts (e.g., UMLS or SNOMED CT) using automated string matching followed by manual review of ambiguous mappings. Disagreements between reviewers can be resolved by consensus among authors.

S1.5 NOTES ON REPRODUCIBILITY

All prompts, task instructions, and scoring rules described in this Supplementary file are designed to mirror the procedures outlined in the Methods section of the main manuscript. Together with the case index and anonymized model outputs in Supplementary File S2, they provide sufficient detail for independent researchers to reproduce the reported results or extend the benchmark with additional metrics.
